# Diagnosis of Cardiac Surgery-Associated Acute Kidney Injury

**DOI:** 10.3390/jcm10163664

**Published:** 2021-08-19

**Authors:** Christina Massoth, Alexander Zarbock

**Affiliations:** Department of Anesthesiology, Intensive Care and Pain Medicine, University Hospital Münster, Albert-Schweitzer-Campus 1, A1, 48149 Münster, Germany; christina.massoth@ukmuenster.de

**Keywords:** cardiac surgery, acute kidney injury, biomarkers, cardiopulmonary bypass

## Abstract

Acute kidney injury after cardiac surgery is characterized by specific patterns of damage and recovery that are important to consider for management and outcome. The Kidney Disease: Improving Global Outcomes (KDIGO) classification covers only part of the conceptual framework and is thus insufficient for a comprehensive diagnosis. This review highlights the strengths and limitations of the recent criteria and provides an overview of biomarkers of cardiac surgery-associated acute kidney injury (CSA-AKI). The evolving understanding of CSA-AKI as a time-sensitive condition has increased the demand to enhance the diagnostic criteria and translate biomarkers into clinical practice.

## 1. Introduction

Acute kidney injury (AKI) is a common but still underrecognized complication, occurring in 10 to 15% of all hospitalized patients and in almost 60% of patients admitted to the intensive care unit [1,2]. Cardiovascular procedures are among the most important risk factors for perioperative renal impairment, and the incidence of AKI following cardiac surgery may be as high as 80% [3]. A large epidemiological study of 3.6 million US veterans found that cardiac surgical procedures have the highest risk of postoperative AKI (relative risk (RR), 1.22; 95% CI, 1.17–1.27) compared to general thoracic surgery (RR 0.92; 95% CI 0.87–0.98), vascular surgery (RR 0.68; 95% CI 0.64–0.71), and other procedures [4]. While mortality rates among patients requiring renal replacement therapy may approach 50%, mild and transient renal dysfunctions also exert an independent effect on the risk of developing chronic kidney disease [3,5]. Since renal replacement therapy remains the only available therapeutic option, risk management relies on prevention and timely diagnosis to implement supportive care as early as possible.

## 2. Pathophysiology of CSA-AKI

While the term “AKI” summarizes a syndrome of acute organ damage and dysfunction with various pathophysiologies, the subset occurring after cardiac surgery is specifically referred to as cardiac surgery-associated acute kidney injury (CS-AKI). Characterizing the different entities of AKI has become increasingly important, since emerging evidence suggests that each type has unique molecular and diagnostic features and requires differentiation in treatment [6].

The majority of patients undergoing cardiac surgical procedures present with a range of predisposing factors and comorbidities, such as advanced age, insulin-dependent diabetes mellitus, heart failure, and chronic kidney disease. It is widely recognized that these conditions are strongly associated with an unfavorable renal outcome and should therefore be addressed by risk assessments [7]. Clinical risk scores, such as the Cleveland Score, the Birnie Score, and the Mehta Score, were developed for patients undergoing cardiac surgery to predict the postoperative probability of AKI and stratify the need for renal replacement therapy [8,9,10].

Exposure of this at-risk population to the noxious stimuli of cardiopulmonary bypass and ischemia-reperfusion injury following aortic cross-clamp might contribute to the development of CSA-AKI. Hypoxemia and the accumulation of metabolic products cause acidosis and cellular damage. Extracorporeal circulation is associated with significant hemodynamic alterations, renal hypoperfusion, and hemolysis [11]. The inflammatory response and development of reactive oxygen species following the reperfusion of hypoxemic tissues further exacerbate cellular dysfunction and injury and stimulate apoptotic mechanisms and recovery pathways [12,13,14]. Recent findings indicate that the renin–angiotensin–aldosterone system is also involved in the development of CSA-AKI, suggesting a reduced activity of angiotensin-converting enzyme (ACE) and decreased levels of angiotensin II associated with cardiopulmonary bypass [15].

## 3. Diagnostic Criteria and the Spectrum of Disease

Several definitions of the condition formerly known as acute renal failure have limited the comparability of clinical outcome measures and the general provision of consensus recommendations. The first standardized definition of changes in kidney function, the RIFLE (Risk Injury Failure Loss End-Stage Renal Disease) classification, was introduced in 2004 and later advanced by the Acute Kidney Injury Network, resulting in the AKIN criteria in 2007 [16,17]. Finally, the Kidney Disease: Improving Global Outcomes Work Group merged the AKIN and RIFLE classifications in 2012 and established the most recent criteria to identify and stage AKI. The KDIGO criteria classify three severity stages and define AKI as an increase in serum creatinine by 1.5 × the baseline amount within the last 7 days or ≥0.3 mg/dL within 48 h, a decline in urine output <0.5 mL/kg/h for 6 h, or both [18].

**Figure 1:**
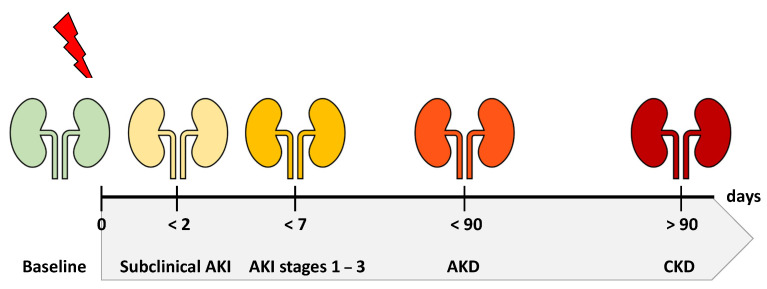
The spectrum of disease: AKI (acute kidney injury), AKD (acute kidney disease), CKD (chronic kidney disease).

New insights have furthered our understanding of AKI as part of a continuum, ranging from a healthy state to chronic kidney disease. The spectrum of this conceptual framework covers subclinical damage preceding KDIGO stage 1 and embeds AKI persisting beyond 7 days within the category of Acute Kidney Disease and Disorders (AKD). While renal recovery in whole or in part may begin at any point within this timeline, the persistence or progression of disease may occur as well, eventually resulting in chronic kidney disease (CKD) after a duration of 3 months (Figure 1) [19,20].

This conceptual change increasingly challenges the biomarkers that are used to define AKI, since serum creatinine and urine output are of limited value in predicting recovery or the progression of disease and even less fit for detecting subclinical damage.

## 4. Serum Creatinine and Urine Output

Traditional renal biomarkers still continue to be the standard of care and remain indispensable for the assessment and monitoring of kidney function. Serum creatinine is ubiquitously accessible and changes are easily recognized and can even be integrated into electronic alerts for automated detection [21,22]. The kinetics of serum creatinine were found to predict adverse outcomes in patients undergoing cardiac surgery, and each small increase of 0.12 mg/dL was associated with increased mortality (OR 1.17 (95% CI 1.11–1.23)) [23].

Although serum creatinine remains the more recognizable criterion, it is vital not to neglect the monitoring of urine output: a large database analysis of 32,045 critically ill patients found even brief episodes of isolated oliguria to be associated with decreased 1-year survival rates [24], although it is unclear whether this also applies to cardiac surgery patients [25]. Oliguria without concomitant changes in serum creatinine is a common finding in patients after cardiopulmonary bypass and affects more than 40% postoperatively [3], but its clinical significance is controversial. A retrospective cohort study of 311 patients undergoing cardiac surgery found no association of isolated oliguria 48 h pos operation with mortality, renal replacement therapy, or time until discharge [26]. By contrast, Priyanka et al. reported from a retrospective analysis of 6637 cardiac surgery patients that stage 1 AKI based on a decreased urine output was associated with an increased risk of a composite endpoint consisting of death, dialysis, and persistent renal dysfunction at day 180 (odds ratio (OR) 1.76; 1.20–2.57; *P* = 0.004). Moreover, 8.1% of patients who presented initially with decreased urine output alone developed chronic kidney disease at day 180, compared to 5.2% of patients without AKI. The authors concluded that a decreased urine output after cardiac surgery is associated with a worse long-term outcome [3].

The intensified monitoring of diuresis was also consistently shown to significantly increase the overall detection of AKI (OR 1.22; *p* < 0.002) in other populations, and was even identified as a measure to improve long-term outcomes. A retrospective analysis including 15,724 patients in the ICU reported it to be associated with less fluid overload (2.49% vs. 5.6%; *p* < 0.001)), lower cumulative fluid volume (2.9l vs. 3.7l; *p* < 0.001), and reduced 1-year mortality rates in patients with AKI (HR 0.9; 95% CI 0.81–0.99; *p* = 0.04) [27].

The diagnostic value of urine output can be further enhanced by a furosemide-stress test. Urine output of less than 200 mL within two hours after the application of furosemide (1.0 or 1.5 mg/kg) predicts the progression of AKI with an area under the curve (AUC) of 0.87 and even outperforms several urinary biomarkers [28,29]. Since the response to furosemide directly relies on tubular integrity and already reflects tubular damage at an early stage, a furosemide challenge could be a useful tool to guide the decision as to whether and when to initiate renal replacement therapy [30].

Another simple option to improve the discriminative value of the traditional markers is their additive application. Since serum creatinine and urine output both represent distinct mechanisms of renal dysfunction, this comprehensive approach was found to identify patients with the most unfavorable outcomes. Kellum et al. reported a comparable risk for RRT and similar 1-year mortality rates in patients who met AKI stage 3 according to urine output or serum creatinine alone (RRT: 2.1% vs. 4.9%; 1-year mortality: 28% vs. 31.9%), with both rising significantly in patients with severe AKI due to a combination of criteria (RRT: 25%; 1-year-mortality: 47.9%) [24].

However, both criteria are recognized to have several shortcomings. As functional markers, they do not indicate incipient damage until the threshold for functional decline is exceeded. Oliguria is not a specific marker of AKI and can occur secondary to the conditions of hypovolemia or renal hypoperfusion [31]. Serum creatinine, in particular, lacks sensitivity and depends as a filtration marker on volume distribution and hyperfiltration. It rises after the renal reserve is exhausted and a functional loss of more than 50% has occurred. Moreover, serum creatinine levels are affected by factors such as age, muscle mass, and dietary intake [32]. Particularly in patients undergoing cardiac surgery, postoperative serum creatinine levels are of limited use, since the priming volume for the extracorporeal circuit contributes to hemodilution and has regularly been shown to cause an initial decrease after surgery [33].

## 5. Biomarkers of CSA-AKI

To date, several biomarkers that can be used to enhance the early diagnosis of CSA-AKI have been identified and extensively investigated. These include biomarkers of tubular damage, such as alkaline phosphatase [34] and N-acetyl-ß-D-glucosaminidase [35]; markers of inflammation, such as interleukin-18 [36,37]; and markers of decreased proximal tube reabsorption, including cystatin c [38], beta-2-microglobulin [39], and retinol-binding protein [40].

### 5.1. NGAL

Neutrophil gelatinase-associated lipocalin (NGAL), expressed by the proximal tubular epithelia and neutrophils, is among the most early and highly upregulated genes after renal injury. Although it was discussed as one of the most specific and sensitive biomarkers for detecting CSA-AKI [41], variable diagnostic performances have been reported [42,43,44,45]. A meta-analysis of 24 studies found an overall sensitivity of 0.68 (95% confidence interval (CI), 0.65–0.70) and a specificity of 0.79 (95% CI, 0.77–0.80). Performance characteristics were found to be superior in children over adults and, in the adult population, in those without a history of CKD [46].

### 5.2. KIM-1

Kidney injury molecule 1 (KIM-1) is a transmembrane glycoprotein with a similar upregulation in the proximal tubule cells after ischemia-reperfusion injury. A subgroup analysis of four trials from a meta-analysis reported a sensitivity of 0.73 (95% CI 0.45–0.93) and a specificity of 0.77 (0.62–0.90) in the cardiac surgery population. However, the diagnostic performance was dependent on factors such as the clinical setting and population and test characteristics, which limited the establishment of a consistent cutoff value that would have defined its clinical importance [47].

### 5.3. [TIMP-2] × [IGFBP7]

To date, the most promising biomarker candidates for CSA-AKI are two molecules that are involved in G_1_-cell-cycle arrest. In an early response to ischemia-reperfusion injury, renal tubular cells enter G_1_-cell-cycle arrest to allow repair and to avoid inflammation and cell death. The product of insulin-like growth factor-binding protein 7 (IGFBP7) and tissue inhibitor of metalloproteinases-2 (TIMP-2) increases as a sign of incipient damage, with a characteristic kinetic peak four hours after cardiopulmonary bypass [48]. With the NephroCheck^®^ test (Astute Medical, San Diego, CA, USA), there is even a point-of-care test to measure [TIMP-2] × [IGFBP7] that is commercially available in several European countries and was approved in 2014 by the Food and Drug Administration in the United States [49]. A systematic review and meta-analysis including 8 studies and a total of 552 cardiac surgery patients reported a reliable performance of [TIMP-2] × [IGFBP7] for the detection of CSA-AKI, with a pooled sensitivity of 0.79 (95% CI, 0.71–0.86, *I*^2^ = 74.2%), a specificity of 0.76 (95% CI, 0.72–0.80, *I*^2^ = 80.8%), and an area under the curve estimated by a summary receiver operating characteristic of 0.868 [50]. Moreover, data from a recent single-center trial including 400 cardiac surgery patients suggested the possibility of detecting subclinical AKI intraoperatively. Cummings et al. found a bimodal elevation of [TIMP-2] × [IGFBP7] with the first peak during surgery in patients who developed subsequent stage 2–3 AKI within 48 h, compared to patients without AKI who presented with no elevation at all [51].

However, [TIMP-2] × [IGFBP7] was also challenged for its variable diagnostic performances. Recently, Grieshaber et al. reported the results from a risk-score-guided observational trial with [TIMP-2] × [IGFBP7] measurements in high-risk patients among a cohort of 613 patients undergoing cardiac surgery. [TIMP-2] × [IGFBP7] was merely predictive of early AKI 24 h after surgery (AUC 0.63; *p* = 0.017), but not of the primary endpoint of AKI stages 2–3 until postoperative day 6 (AUC 0.58; *p* = 0.26) [52].

## 6. Novel Candidates

### 6.1. Dickkopf-3

While the present biomarkers are still under validation for implementation in clinical practice, there are already several new emerging candidates to detect and guide the management of CSA-AKI. Among these, urinary Dickkopf-3 (DKK3), a modulator of tubulointerstitial fibrosis, was identified as a marker of ongoing tubular stress. Preoperative urinary levels of DKK3 detected patients at risk and predicted postoperative AKI with an AUC of 0.783 ((95% CI 0.747–0.820), *p* < 0.0001). Additionally, high preoperative levels were associated with significantly reduced long-term kidney function and were reflective of AKI-to-CKD transition [53].

### 6.2. CCL-14

Urinary C-C-motif chemokine ligand 14 (CCL-14) was recently introduced as a predictor of persistent AKI and renal replacement therapy in a patient cohort with stage 2–3 CSA-AKI. CCL-14 is released from tubular epithelial cells as an inductor of chemotaxis and monocyte-macrophage recruitment in response to renal injury. Although it is not a marker for early diagnosis, it provides diagnostic information on the anticipated severity and progression of disease.

CCL-14 was found to predict persisting AKI after cardiac surgery with an AUC of 0.930 (95% CI, 0.881–0.979) and renal replacement therapy within 7 days with an AUC of 0.915 (95% CI, 0.858–0.972). Besides these discriminative properties that can be used to differentiate patients with persistent AKI from those with renal recovery, its performance remained unaffected by chronic comorbidities and CKD [54,55].

### 6.3. Renin

The renin–angiotensin–aldosterone system (RAAS) is a complex hormone-signaling pathway that plays an essential role in regulating blood pressure and electrolyte and fluid balance. The activation of the RAAS in response to decreased blood flow, blood volume, or sodium levels stimulates the secretion of renin by juxtaglomerular cells to activate in several steps the active metabolite, angiotensin II [56]. In a cohort of patients undergoing cardiac surgery, higher renin levels 4 h after cardiopulmonary bypass were associated with AKI. Compared to renin levels, [TIMP-2] × [IGFBP7] or urinary DKK3, the changes between pre-and postoperative renin levels (Δ-renin) performed best in predicting postoperative AKI with an AUC of 0.817 (95% CI, 0.747–0.887) [15].

### 6.4. Free Hemoglobin

Intravascular hemolysis is a well-known complication of extracorporeal circulation. The corresponding release of free hemoglobin (fHb) from the red blood cells has been recognized as a trigger of oxidative stress and promotor of AKI. A secondary analysis of a randomized controlled trial identified a ratio > 2.23 of fHb at the end of cardiopulmonary bypass to baseline pre-surgery levels as predictive for the development of AKI (80.0% vs. 49.1%; *P* = 0.001) and the need for renal replacement therapy (10.9% vs. 0%; *P* = 0.036). With the highest AUC of 0.704 (95% CI 0.592–0.804), fHb outperformed other biomarkers, such as NAG, NGAL, and KIM-1, that were used for comparison in this analysis [57].

## 7. Biomarker-Enhanced Diagnostic Criteria?

Although these novel markers seem to be promising candidates for enhancing the diagnostic capacities for CSA-AKI, there is still a need for further validation studies. However, since increasing evidence is showing that CSA-AKI is not an unchangeable fate but can be prevented and mitigated, it is increasingly urgent to translate biomarkers into clinical practice [58,59].

Recently, the results of the PrevAKI multicenter trial confirmed once again that the [TIMP-2] × [IGFBP7]-guided implementation of a simple care bundle consisting of the optimization of volume status and hemodynamics, functional hemodynamic monitoring, the avoidance of nephrotoxic drugs, and the prevention of hyperglycemia effectively reduced the incidence of stage 2 and 3 AKI in comparison to the control group (14.0% vs. 23.9%; ARR 10.0% (95% CI, 0.9–19.1); *P* = 0.034) [60]. This trial confirmed previously published trials showing that an individualized approach guided by biomarkers prevents the development of AKI [58,61,62]. Consequently, it is apparent that it is time to reconsider the recent diagnostic criteria.

**Figure 2:**
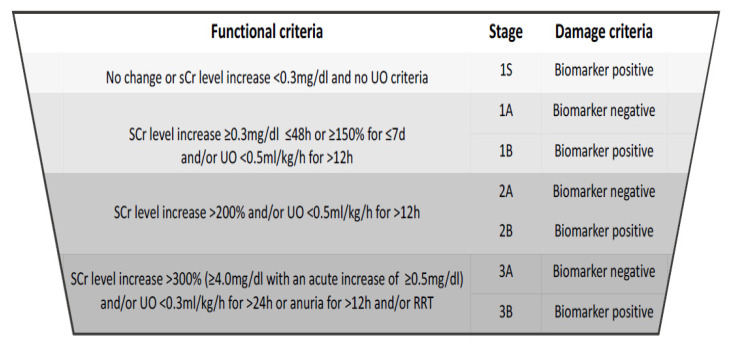
New, biomarker-enhanced definition of acute kidney injury according to the Acute Disease Quality Initiative Consensus Conference proposal, modified from Ostermann et al.

A consensus statement by the Acute Disease Quality Initiative Consensus Conference on the implementation of biomarkers in clinical routine to prevent, diagnose, and manage AKI proposed an advanced staging system based on biomarkers of function and damage [63]. In this modification, the three KDIGO stages were subcategorized into a biomarker-negative (1A, 2A, 3A) and a biomarker-positive (1B, 2B, 3B) subgroup. Stage 1 was further divided into a third category (1S), reflective of subclinical damage and defined solely as biomarker-positive without the serum creatinine and urine output criteria to be met [63]. Although there are still considerable limitations on and knowledge gaps within the application of biomarkers, opening up the diagnostic criteria to their inclusion will highlight the particular pathophysiologic pathways of damage. A more differentiated diagnosis will likely be able to improve the management of CS-AKI in particular, potentially providing the basis for more individualized care (Figure 2).

## 8. Conclusions

Cardiac surgery-associated AKI features specific patterns of damage and recovery that go unrecognized by recent diagnostic criteria. The role of timing as a crucial factor in the management of CS-AKI demands enhanced criteria reflective of subclinical damage. Biomarkers of CS-AKI provide additional discriminative features and are on the threshold of finding their way into clinical practice.

## Data Availability

Not applicable.
